# Prognostic factors for recurrence and malignant transformation after treatment of oral epithelial dysplasia: A mixed cohort study

**DOI:** 10.1371/journal.pone.0337402

**Published:** 2025-12-19

**Authors:** Zohreh Moradipour, Samira Derakhshan, Monir Moradzadeh Khiavi, Nafiseh Sheikhbahaei, Mohamad Javad Kharazifard

**Affiliations:** 1 School of Dentistry, Tehran University of Medical Sciences, Tehran, Iran; 2 Department of Oral and Maxillofacial Pathology, School of Dentistry, Tehran University of Medical Sciences, Tehran, Iran; 3 Department of Oral & Maxillofacial Medicine, School of Dentistry, Tehran University of Medical Sciences, Tehran, Iran; 4 Dental Research Center, School of Dentistry, Tehran University of Medical Sciences, Tehran, Iran; University of Hong Kong, HONG KONG

## Abstract

**Purpose:**

Oral epithelial dysplasia (OED) is a premalignant condition with a variable risk of malignant transformation (MT). This study investigates the recurrence and MT rates in an Iranian patient cohort with OED, evaluating associations with demographic factors, lifestyle habits, and histopathologic grading systems, with the aim of providing population-specific prognostic insights and enhancing disease prediction and patient outcomes.

**Methods:**

This mixed cohort study included 89 surgically treated patients with OED, followed for 24–168 months. Demographic data, smoking and alcohol consumption history, recurrence, and MT status were collected from medical records. Histopathological grading was performed using both the WHO 2017 classification and a binary grading system. Kaplan-Meier survival analysis, Cox regression, and statistical tests were applied to identify prognostic factors. A p-value <0.05 was considered statistically significant.

**Results:**

During follow-up, 22.5% (20/89) of patients experienced recurrence, and 30.3% (27/89) underwent MT. The tongue was the most common site for OED (48.3%), recurrence (55%), and MT (44.4%). Males were more frequently affected than females (60.7% vs. 39.3%,), but females tended to remain cancer-free more often. Smoking and alcohol consumption did not significantly influence cancer development.

Kaplan-Meier analysis showed 50% of MT cases occurred after about 108 months (mean: 114.8 months). The combined mean survival for recurrence and MT was 92.8 months. Cox regression identified WHO grading as the only statistically significant predictor of MT and recurrence/MT (p = 0.001).

**Conclusions:**

The WHO grading system plays a crucial role in predicting the likelihood of OED recurrence and malignant transformation. Lesion location showed only a non-significant trend, suggesting that its effect may be mediated by grade. These findings highlight the importance of using grading-based risk assessments and ensuring close follow-up care to better manage OED and improve patient outcomes.

## 1. Introduction

Oral epithelial dysplasia (OED) is a premalignant condition characterized by abnormal histopathological changes in the oral epithelium, increasing the risk of developing oral squamous cell carcinoma (OSCC) [1,2].

The main etiological factors for OED and oral carcinoma are smoking and alcohol use [2]. OED can be a histopathologic feature of oral potentially malignant disorders (OPMDs) such as leukoplakia, erythroplakia, or leukoerythroplakia [2]. Histological evaluation for the presence of OED is considered the standard practice for predicting malignant transformation (MT) in these lesions [1]. Factors such as gender, age, grade of dysplasia, lesion location, lesion size, clinical features, and alcohol and tobacco consumption play a role in the development of MT in lesions such as leukoplakia. A history of systemic diseases, regardless of treatment, also contributes [2]. Most MTs occur in lesions with a history of recurrence; however, some cases transform without ever recurring [3,4].

While a precise, universally accepted global incidence of OED is not available due to its histopathological nature, the epidemiology of OPMDs provides a crucial context. The average prevalence of OPMD in the general population ranges from 1% to 5% [5]. For instance, in India, the overall prevalence was reported to be 13.28% [6]. This variation is strongly linked to different etiological factors, with prominent risk factors in Western countries being smoking and alcohol drinking, while in Asian countries, smokeless tobacco consumption and betel quid chewing are more significant [7]. A community-based study in North Eastern Thailand reported that individuals with a betel quid chewing habit had a 5.12-fold increased probability of developing OPMDs [8]. MT rate of OPMDs is also highly variable, with a systematic review and meta-analysis showing an average rates varying from 0.13% to 34% [9]. The risk of malignant change differs significantly between OPMD subtypes, with reported rates of 9.5% for leukoplakia, 33.1% for erythroplakia, 1.4% for oral lichen planus, and 5.2% for oral submucous fibrosis [10].

Geographic differences have also been noted, with higher transformation rates reported in Southeast Asia compared to Europe and North America, likely reflecting differences in environmental exposures and lifestyle factors [8,9].This wide variation can be attributed to differences in follow-up durations, study group definitions and selection criteria, geographical disparities, and smoking/alcohol consumption patterns [11–13]. Although the risk of MT persists in patients with OED, those undergoing surgical interventions demonstrate lower rates of malignant changes and recurrence compared to individuals receiving non-surgical treatment modalities [14]. Previous studies have reported that the rate of malignant changes arising from OED ranges between 1.4% and 36% [10,15–17]. A meta-analysis by Mello et al. further indicated a pooled MT rate of 10.5% among histologically confirmed OED cases [18]. Other studies reported lower progression rates, with less than 5% of OED cases undergoing transformation and an estimated annual MT rate of ~1% [19], while another investigation found an annual MT rate of 1.7% in mild OED, with substantially higher rates in severe lesions [20]. Longitudinal cohort data have shown that, after excluding early events (<12 months), OED may exhibit an annual MT rate of 6.4%, with corresponding reductions in mean time to malignant progression [21]. In addition, broader population-based studies have reported MT rates as high as 22.9%, with relatively stable annual transformation rates between 4.5% and 6.3% over more than a decade of follow-up [22].

Studies have shown a higher likelihood of malignant changes and recurrence in elderly patients compared with younger patients [23]. Moreover, the rate of MT in OED varies [10]. While higher OED grades are predominantly associated with increased MT risk [23–25], some studies haven’t found a clear correlation between MT rate and grade [2,26]. Geographical location may also influence transformation rates [27,28]. Following surgical excision, the risk of MT exists due to the field cancerization theory [29–32]. However, fully excised lesions have lower MT rates than incompletely removed ones [20]. Periodic follow-up of OED lesions can lead to early detection of MT, and improved prognoses for patients with OSCC [10,33].

While existing research has evaluated the role of influencing factors in the MT of OPMDs, a significant knowledge gap remains, particularly concerning the Iranian population. Studies have shown that the factors contributing to MT of OPMDs vary based on geographical region and lifestyle differences. For example, female patients in a southern Iran study with MT of leukoplakia were mostly non-smokers (76.4%), while males with the same condition were mostly smokers (63.8%). This highlights a distinctive pattern compared to many other populations where male and female smoking habits are more similar as a risk factor [34].

Furthermore, while most previous studies have focused on OPMDs, which are clinical diagnoses, our research addresses the more advanced, histopathological diagnosis of OED. We are also concurrently investigating both the recurrence and MT of these surgically excised lesions, an area with limited existing research.

This study aimed to investigate the rate of recurrence and MT of treated OED lesions by surgical excision in the Iranian population and their relation to demographic factors, smoking habits, alcohol consumption, and OED grading. By evaluating the impact of these factors on OED progression, we seek to enhance disease prediction, inform clinical decision-making for treatment and follow-up, and ultimately improve patient care, quality of life, and reduce mortality.

## 2. Materials and Methods

### 2.1 Study design and aim

This mixed cohort (retrospective-prospective) study was conducted at the Faculty of Dentistry, Tehran University of Medical Sciences. Patients diagnosed with OED between 2008 and early 2022 were retrospectively identified from archived medical records in the Department of Oral Pathology, and their histopathological diagnoses were verified. Then follow-up data regarding recurrence and MT were prospectively collected between 20 February and 19 April 2024.

The primary aim was to identify factors associated with recurrence and MT—such as demographic characteristics, histopathologic grading, smoking habits, and alcohol consumption—in order to improve disease prediction and patient outcomes.

### 2.2 Inclusion criteria

- Histopathologically confirmed diagnosis of OED
- Underwent surgical excision
- Availability of complete demographic and clinical data

### 2.3 Exclusion criteria

- Presence of syndromes or systemic diseases affecting the mucosal surface
- Positive dysplastic margins
- History of head and neck cancers

### 2.4 Data collection

Demographic data, smoking history, alcohol consumption, and clinical outcomes were gathered from medical records and follow-up visits. All cases included in our study had a histopathologically confirmed diagnosis of Oral Epithelial Dysplasia (OED) in their previous medical records. For the final outcome assessment, both recurrence and MT were confirmed via histopathological evaluation. All clinically suspicious cases identified during follow-up were subject to a confirmatory biopsy, ensuring that a diagnosis of recurrence or MT was based exclusively on pathological evidence.

To evaluate OED, two grading systems were employed:

1. **WHO 2017 Grading Classification**: A standard histopathological grading system used to categorize dysplasia into mild, moderate, or severe.
2. **Binary Grading System**: Lesions were classified as low-grade dysplasia (if fewer than 4 structural criteria or 5 cellular criteria were met) or high-grade dysplasia (if these thresholds were exceeded), as described by Kujan et al. (2006) [35].

All histopathological slides were independently reviewed by two experienced pathologists in separate, blinded sessions to minimize observer bias.

For smoking and alcohol consumption, we considered *current smoker* and *current drinker* status. A *current smoker* was defined as an individual who self-reported smoking cigarettes, pipes, or cigars within the calendar year prior to the year of diagnosis. Similarly, a *current drinker* was defined as a person who self-reported consuming any type of alcoholic beverage within the calendar year prior to the year of diagnosis [28]. In addition, individuals who reported smoking more than 100 cigarettes or the equivalent amount of tobacco products, and those who reported consuming an average of five or more alcoholic beverages per week during that same period, were classified as current smokers or drinkers.

#### 2.4.1 Sample size and power analysis.

A priori power analysis was performed to ensure adequate sample size. Based on an expected MT rate of 25% and a power of 80% (α = 0.05), a minimum of 84 patients was required. The final sample of 89 patients exceeded this threshold, supporting the robustness of the analyses.

### 2.5 Statistical analysis

Descriptive statistics, Cox regression, Kaplan-Meier, cross-tabs, post-hoc power analysis and One-way ANOVA analyses were conducted using SPSS software v.25.0 to investigate the association between recurrence and MT in OED with demographic characteristics and two key risk factors (smoking and alcohol consumption). A *p-value*<0.05 was considered statistically significant.

### 2.6 Ethical considerations

This study was approved by the Ethics Committee of the School of Dentistry, Tehran University of Medical Sciences (Ethics code: IR.TUMS.DENTISTRY.REC.1401.059). All participants were adults (≥18 years old) at the time of initial contact and were eligible to provide consent. Verbal informed consent was obtained via telephone before the follow-up, and this procedure was approved by the Ethics Committee due to the non-invasive nature of the study and because the follow-up was part of routine care. The verbal consent process was documented through clinical logs. Only individuals who agreed to participate were included in the study. Written consent was not required. The patients’ information was reviewed and coded by one of the authors (ZM) and used in a confidential and anonymized manner for research purposes.

## 3. Results

This study monitored 89 patients with OED who underwent surgical excision for a period of 24–168 months.

Table 1 summarizes the distribution of 89 patients with oral epithelial dysplasia (OED) after treatment.

**Table 1:** Descriptive characteristics of OED Patients % (n).

Characteristic	% (n)
Gender	
Male	60.7% (54)
Female	39.3% (35)
Total	100% (89)
Smoking	
Yes	37.1% (33)
No	62.9% (56)
Total	100% (89)
Alcohol consumption	
Yes	16.9% (15)
No	78.7% (70)
Missing	4.5% (4)
Total	100% (89)
Location	
Tongue	48.3% (43)
Buccal mucosa	14.6% (13)
Floor of the mouth	9.0% (8)
Other areas	28.1% (25)
Total	100% (89)
WHO grading	
Mild	40.4% (36)
Moderate	43.8% (39)
Severe	15.7% (14)
Total	100% (89)
Binary-grading	
Low-grade	62.9% (56)
High-grade	37.1% (33)
Total	100% (89)
Clinical appearance	
White-Plaque	47.2% (42)
White-red plaque	15.7% (14)
Verrucous surface	11.2% (10)
Plaque with ulcer	9.0% (8)
Other	16.9 (15)
Total	100% (89)

Table 2 categorizes patients by their disease state (disease-free, recurrence, or MT). It reveals correlations between disease state and various variables including demographics (age, gender, and location), lifestyle habits (smoking and alcohol consumption), clinical appearance, and OED grading systems (WHO 2017 grading and binary grading).

**Table 2:** Distribution of OED patients by disease state (disease-free, recurrence, MT) after treatment and its correlations with demographics (age, gender, location), habits (smoking, alcohol), and OED grading (WHO 2017, binary) % (n).

State	Disease-free	Recurrence	MT	Total	p-value
	%(n)	%(n)	%(n)	%(n)	
Number	47.2% (42)	22.5% (20)	30.3% (27)	100% (89)	
Range (Mean) age	26-83 (58.05)	41-78 (60.75)	33-88 (67.46)	26-88 (61.40)	0.135
(year)					
Gender					
Male	42.6% (23)	25.9% (14)	31.5% (17)	100% (54)	0.712
Female	54.3% (19)	17.1% (6)	28.6% (10)	100% (35)	
Smoking					
Yes	51.5% (17)	30.3% (10)	18.2% (6)	100% (33)	
No	46.6% (25)	17.9% (10)	37.5% (21)	100% (56)	0.907
Alcohol consumption					
Yes	66.6% (10)	26.7% (4)	6.7% (1)	100% (15)	
No	42.9% (30)	20.0% (14)	37.1% (26)	100% (70)	0.202
missing	38.5% (2)	53.8% (2)	0.0% (0)	100% (4)	
WHO grading					
Mild OED	80.6% (29)	8.3% (3)	11.1% (4)	100% (36)	0.001
Moderate OED	30.8% (12)	38.5% (15)	30.8% (12)	100% (39)	
Severe OED	14.3% (2)	14.3% (2)	71.4% (10)	100% (14)	
Binary-grading					
Low-grade	60.7% (34)	21.4% (12)	17.9% (10)	100% (56)	0.196
High-grade	24.2% (8)	24.2% (8)	51.5% (17)	100% (33)	
Clinical appearance					
White-Plaque	54.8% (23)	21..4% (9)	23.8% (10)	100% (42)	0.45
White-red Plaque	35.7% (5)	14.3% (2)	50.0% (7)	100.0% (14)	
Verrucous surface	50.0% (5)	30.0% (3)	20.0% (2)	100% (10)	
Plaque with ulcer	25.0% (2)	62.5% (5)	12.5% (1)	100% (8)	
Others	46.6% (7)	6.6% (1)	46.6% (7)	100.0% (15)	
Total	47.2% (42)	22.5% (20)	30.3% (27)	100.0% (89)	
Location					
Tongue	46.5% (20)	25.6% (11)	27.9% (12)	100% (43)	0.087
Buccal mucosa	46.1% (6)	23.3% (3)	30.6% (4)	100% (13)	
Floor of the mouth	25.0% (2)	25.0% (2)	50.0% (4)	100% (8)	
Other areas	56.0% (14)	16.0% (4)	28.0% (7)	100% (25)	

OED: Oral Epithelial Dysplasia, MT: Malignant transformation.

**Fig 1:**
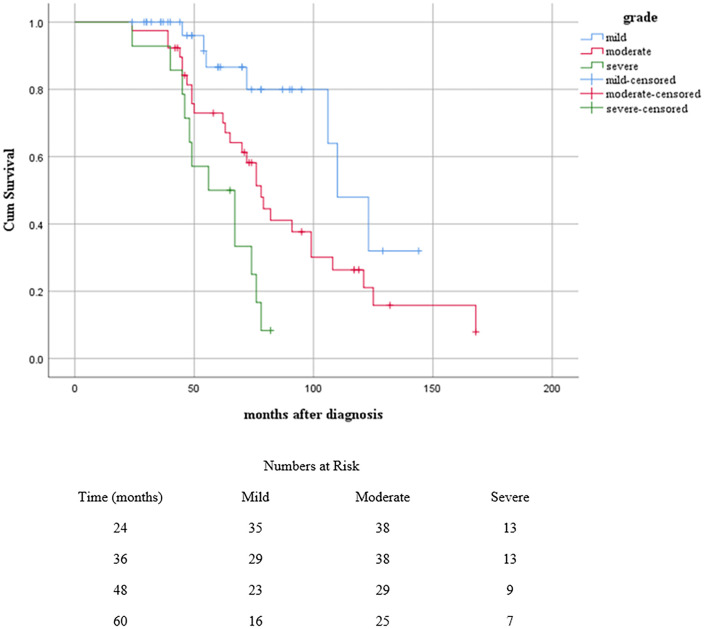
Kaplan-Meier survival curves showing the cumulative probability of patients remaining free of MT or recurrence based on WHO grading (mild, moderate, and severe). The cross marks (+) indicate censored cases. The table below the curve shows the number of patients at risk in each grading group at specified time intervals.

**Fig 2:**
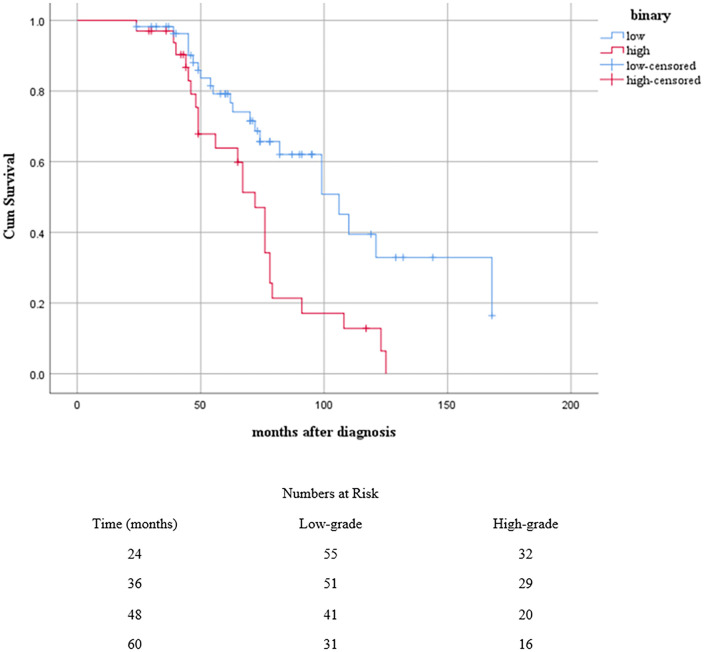
Kaplan-Meier survival curves showing the cumulative probability of patients remaining free of MT or recurrence based on binary grading (Low-grade and High-grade). The cross marks (+) indicate censored cases. The table below the curve shows the number of patients at risk in each grading group at specified time intervals.

Survival data analysis (Kaplan-Meier test) showed that 10% of MTs occurred after 30 months, and 50% of MTs happened after about 108 months with a mean of 114.8, and a median of 121.0 months, and survival data analysis for both recurrence and MTs showed a mean of 92.8, and median of 78.0 months. For enhanced clarity, a “Number at Risk” table has been added below each figure to show the number of patients followed at various time points. *(*Figs 1 and 2*).*

A post-hoc power analysis was conducted to evaluate the study’s ability to detect significant associations between clinical and histopathological factors and MT. For Cox regression analysis, we used the observed sample size (N = 89), the event rate (30.3% of patients experienced MT), and the hazard ratios (HRs) for the key variables. The hazard ratio for WHO grading was HR = 3.028, and for location, HR = 1.412. Using a significance level (α = 0.05), we estimated the statistical power for each association.

Cox regression analysis was performed on all variables—including age, gender, lesion location, smoking status, alcohol consumption, and OED grading (WHO 2017 and binary grading systems)—to evaluate their impact on MT and the combined outcome of recurrence and MT. The results revealed that WHO grading was the only statistically significant predictor for MT and recurrence/MT (p = 0.001). Post-hoc power analysis demonstrated that the study had approximately 87%–90% power to detect the association between WHO grading and MT, and approximately 65%–70% power to detect the association between lesion location and MT.

Cox regression analysis adjusted for age, gender, lesion location, smoking, alcohol consumption, and WHO grading revealed that WHO grading was the only statistically significant predictor of MT and combined recurrence/MT (HR = 2.818, p = 0.001).

According to survival data analysis, a 90% rate of disease-free cases was demonstrated after about 30 months, while the disease-free cases rate of 50% was observed after nearly 80 months.

The rate of recurrence and MT is described based on the degree of dysplasia in both the WHO and binary grading systems as shown in Table 3.

**Table 3:** Disease-free, recurrence, and MT in OED Patients by WHO and binary grading systems.

Histopathological Grading	Total n(%)	Disease-free n(%)	Recurrence n(%)	MT n(%)
WHO grading				
Mild	40.5 (36)	69 (29)	15 (3)	14.8 (4)
Moderate	43.8 (39)	28.6 (12)	75 (15)	44.5 (12)
Severe	15.7 (14)	2.4 (1)	10 (2)	40.7 (11)
Binary grading				
Low grade	62.9 (56)	80.9 (34)	60 (12)	37 (10)
High grade	37.1 (33)	19.1 (8)	40 (8)	63 (17)
Overall	100 (89)	100 (42)	100 (20)	100 (27)

The WHO grading system for OED was compared to a binary grading system. The majority (83.3%) of mild cases using the WHO system were classified as low-grade in the binary system. While the remaining 16.7% were classified as high-grade. Similarly, 61.5% of moderate cases, were considered low-grade in the binary system and 38.5% were high-grade. The majority (85.7%) of severe cases in the WHO grading system were high-grade in the binary system. Only a small portion (14.3%) of severe cases were classified as low-grade.

Conversely, within the low-grade binary grading system, 53.6% were classified as mild, 42.8% as moderate, and 3.6% as severe OED by the WHO system. Meanwhile, only 18.1% of high-grade binary cases are classified as mild, 45.5% as moderate, and 36.4% as severe OED by the WHO grading system.

## 4. Discussion

This study assessed the recurrence and MT rates of OED lesions after surgical treatment in an Iranian population, evaluating associations with demographic factors, lifestyle habits, and grading systems. While previous research has explored factors influencing MT, fewer studies have examined their link to recurrence. OED is a premalignant condition characterized by abnormal histopathologic alteration in the epithelial lining of the mouth [20,35,36]. It carries varying risks of progression to oral cancer. The severity of dysplasia, while often linked to malignancy, does not always predict the outcome, as non-dysplastic lesions can also progress to cancer [37]. Grading dysplasia remains challenging due to its subjective nature and inter-observer variability [1,38]. The WHO grading system classifies OED into three levels [37], whereas the binary grading system simplifies this to low- and high-grade categories, aiding treatment decisions [35].

Our study found a wide age range for OED patients (26–88 years), with an average age of 61.4 years. Patients experiencing MT were older (67.46 years) compared to those remaining disease-free (58.05 years) or experiencing recurrence (60.75 years). These findings align with some previous studies [2,16]. However, the overall age distribution varies across studies, with reported peak prevalence between the third to seventh decades, and a mean age ranging from 49.7 to 54 years [4,17,24,26,39]. Some studies suggest most MT and recurrences occur in patients older than 75 years [23], while others report a majority of cancers in patients around 66–68 years or older [2,20,40].

The impact of age on the recurrence and MT of OED is a subject of debate. While some studies, such as those by Luigi Lorini et al. [41] and Pereira et al. [42], have shown a peak in recurrence in middle-aged individuals, others, like Silverman et al. [39], have not found a significant association between age and cancer development. These discrepancies may be attributed to differences in study populations, such as the inclusion of betel-quid chewers, and lifestyle factors like smoking and alcohol consumption, which can influence the risk of MT at younger ages [43].

Our study observed a higher prevalence of OED in males for initial occurrence, recurrence, and MT, which aligns with most previous research [2,4,16,23,26,43,44]. However, some studies reported a higher prevalence in females or no significant differences between genders [2,40]. Inconsistencies exist regarding MT rates as well, with some studies reporting higher rates in females and others in males [20,26], while others found the opposite [12,17] or some found no significant difference between genders [2,4]. In a study by Gilvetti, both recurrence and MT were higher in women, but the difference was not significant [40]. Although some studies have reported that female sex is associated with shorter carcinoma-free survival [41], in our cohort, females were more likely to remain cancer-free. This discrepancy may reflect differences in study populations, regional risk factors, and sample sizes, highlighting the importance of contextual interpretation of gender-related outcomes.

The diverse distribution of primary sites for OED underscores the complexity and variability of these lesions. While our study and several others have identified the tongue as the most common location for OED [2,20,23,26,40,45] different studies have found buccal mucosa [4,39,43,44,46], alveolar ridge [42], mouth floor [16], and lower lip [47] as primary sites. Although Cox regression did not show a statistically significant effect of location (p = 0.087), a trend was observed. This non-significant result should be interpreted with caution, as a post-hoc power analysis indicated that the study’s power to detect the association between lesion location and MT was lower, at approximately 65–70%. This contrasts with the high power (87–90%) to detect the significant association between WHO grading and MT, reinforcing that while our sample size was sufficient for our primary finding, it may have been a limitation for secondary variables.

The tongue was the most common location for OED, and it was the most common site for recurrence. While the floor of the mouth had a higher incidence of MT. Previous studies similarly report the tongue as a common site for both OED and MT [2,20,23,26]. However, Gillette et al.‘s study found no significant difference between lesion location and recurrence, with MT being more prevalent in the alveolar ridge [40].

Our study revealed a higher prevalence of mild and moderate (low-grade) OED (43.8% and 40.4%) compared to severe (high-grade) cases (15.7%). This suggests that mild and moderate (low-grade OED is more common than severe (high-grade OED, implying that many lesions are diagnosed and treated in their early stages. Previous research supports this finding, reporting a higher prevalence of mild dysplasia [2,16,23,42,43,46]. However, it’s worth noting that a study by Gilvetti et al. exclusively focused on high-grade lesions [40].

The relationship between OED grade and the risk of recurrence and MT is complex and varies across studies. Some studies have shown a correlation between high-grade OED and increased risk of recurrence or MT [4,10,17,20,24]. However, other studies have found that moderate OED may also have a significant risk of recurrence and MT [23,26], while others have not found a significant correlation between grade and MT [2]. These conflicting findings highlight the need for further research to better understand the factors influencing the clinical course of OED.

The recurrence rate (22.5%) and MT rate (30.3%) observed in our study highlight the seriousness of OED, with nearly half of patients experiencing either outcome. A systematic review reports that a wide range of MT rates observed across studies, are influenced by factors such as smoking, alcohol use, and grading inconsistencies [24]. Factors such as demographic differences, lifestyle habits, grading inconsistencies, surgical techniques, margin status, and follow-up duration can influence these rates.

The impact of smoking and alcohol consumption on the progression of OED is complex and not fully understood. In our study the results were not statistically significant. Other studies have shown conflicting results, with some suggesting that non-smokers and non-drinkers may have a higher risk of cancer [2,39], while others have linked smoking and alcohol consumption to increased risk [40]. Another study found the highest recurrence rates in tobacco or betel quid users [4]. Previous studies similar to ours have not incorporated pack years (PY) as a standardized measure of smoking exposure [2,33,34]. This methodological limitation may contribute to the variability and conflicting results observed across studies examining the relationship between smoking and OED. Incorporating PY in future research would help resolve these discrepancies and enhance the reliability of clinicopathological correlations.

This study followed patients with OED for 24–168 months. Previous studies reported follow-up periods varying from 12 months to 30 years [2,4,16,26,39–41,43]. Our study observed a delayed pattern of MT. While previous studies reported a shorter timeframe for MT (30–90 months) [2,16,20,26,39–41,43], our study found that 50% of patients experienced MT after approximately 108 months. The broader range of OED severities included in this study likely contributed to the delayed MT timeframe.

Our study underscores the prognostic significance of the WHO grading system in predicting disease progression, recurrence, and MT in OED. Cox regression analysis identified the WHO grading system as a more accurate predictor of outcomes, with higher grades linked to elevated recurrence and MT risks. However, the binary low-grade category’s broad inclusion of WHO-classified mild (53.6%), moderate (42.8%), and severe (3.6%) cases suggests potential underestimation of severity. Similarly, 45.5% of moderate cases being classified as high-grade in the binary system highlights limitations in its precision.

While the binary grading system offers simplicity and utility in identifying high-risk patients, as supported by Câmara et al. [48] and Odell et al. [49], it appears less effective than the WHO system in distinguishing between low-grade lesions with varying degrees of dysplasia. These findings align with de Freitas Silva et al.‘s conclusion that the WHO grading system provides reliable prognostic information [50]. However, the subjectivity of grading, particularly in moderate dysplasia, remains a challenge across both systems, as highlighted by Kujan et al. Moderate dysplasia often demonstrates overlapping features of low- and high-grade lesions, leading to variability in classification and prognostication [35]. Thus, while binary systems may offer better interobserver agreement [35,48,49], the precise differentiation provided by the WHO system continues to be valuable in subtle clinical decision-making [50].

Additionally, our findings indicated that histopathologic grade (according to WHO 2017 criteria) is a significant factor in predicting patient prognosis. The post-hoc power analysis confirmed that the study had sufficient power (87%−90%) to detect the significant association between WHO grading and MT, supporting the strength of this result. However, the power to detect the association between location and MT was lower (approximately 65%−70%), which may have contributed to the marginal p-value (p = 0.063) observed for this variable. This highlights a limitation of the current study in identifying smaller effect sizes.

The contradictory findings reported across studies regarding gender differences and the prognostic utility of grading may be explained by several factors. Regional variations in patient populations, such as differences in lifestyle and exposure to risk factors, may influence outcomes and account for variability between studies. In addition, methodological differences, including study design, diagnostic criteria, and grading systems, can lead to inconsistencies in reported results. Finally, the limited sample sizes in many studies, including ours, may reduce statistical power and contribute to inconsistent findings. Taken together, these factors suggest that discrepancies in the literature should be interpreted cautiously, and underscore the need for larger, multi-center studies with standardized methodologies to clarify these associations.

Another limitation is related to follow-up duration, which varied between 24 and 168 months. Given the median time to MT was 108 months, the relatively short follow-up duration for some patients may have led to an underestimation of MT rates. However, only a small number of patients (n = 3) had a minimum follow-up of 24 months. The majority of the cohort was followed for a significantly longer period, with the median time to MT occurring more than 9 years after initial treatment. Future studies should ensure longer and more uniform follow-up periods to provide more reliable survival estimates.

## 5. Conclusions

Our study revealed that a significant percentage of patients experienced recurrence and MT, which highlights the importance of OED and the need for close follow-up. The WHO grading system appeared as the most important predictor of recurrence and MT together. While lesion location showed a non-significant trend, our data suggests its effect may be mediated by grade. Moreover, the tongue remained the most common site for OED and recurrence. On the other hand, smoking and alcohol do not significantly affect the risk of MT. These findings underscore the need for further research using prospective data collection methods to explore the influence of lifestyle factors on clinical outcomes.
